# Using cone beam CT to assess the upper airway after surgery in children with sleep disordered breathing symptoms and maxillary-mandibular disproportions: a clinical pilot

**DOI:** 10.1186/s40463-017-0204-4

**Published:** 2017-04-11

**Authors:** Noura A. Alsufyani, Michelle L. Noga, Manisha Witmans, Irene Cheng, Hamdy El-Hakim, Paul W. Major

**Affiliations:** 1 School of Dentistry, Faculty of Medicine and Dentistry, University of Alberta, Edmonton, Canada; 2 Department of Radiology and Diagnostic Imaging, Faculty of Medicine and Dentistry, University of Alberta, Edmonton, Canada; 3 Department of Computing Science, Faculty of Science, University of Alberta, Edmonton, Canada; 4 Department of Surgery, Faculty of Medicine and Dentistry, University of Alberta, Edmonton, Canada; 5 Edmonton Clinic Health Academy- School of Dentistry, 5th floor, 11405-87 AVE NW, Edmonton, T6G 1C9 AB Canada; 6 Department of Oral Medicine and Diagnostic Sciences, College of Dentistry, King Saud University, Riyadh, Saudi Arabia

**Keywords:** Adenoidectomy, Tonsillectomy, Treatment outcome, Cone-beam computed tomography

## Abstract

**Background:**

The surgical excision of anatomic obstructions such as adenoids, palatine or lingual tonsils are commonly performed in children with sleep disordered breathing (SDB). Imaging studies measuring airway changes post-surgery in the SDB pediatric population are scarce, rarely addresses the nasal cavity, and are based on global measures (e.g. volume) that do not represent the complexity of the upper airway anatomy. The purpose of this pilot is to test the feasibility in using cone beam CT (CBCT) to analyze the nasal and pharyngeal airway space post-surgery using meaningful methods of analyses, and correlating imaging findings with clinical outcomes in children with SDB symptoms and maxillary-mandibular disproportion.

**Methods:**

Twelve non-syndromic children with SDB symptoms and jaw disproportions were evaluated by interdisciplinary airway team before and after upper airway surgery. CBCT and OSA-18 quality of life questionnaire pre and post-operatively were completed. Conventional and new airway variables were measured based on 3D models of the upper airways and correlated with OSA-18. Conventional measures include volume, surface area, and cross-sectional area. New airway measures include constriction and patency; point-based analyses.

**Results:**

Eight females and four males were 8.8 ± 2 years with mean BMI of 18.7 ± 3. OSA-18 improved, median (lower quartile-upper quartile) from 64.2 (54.7–79.5) to 37.6 (28.7–43) postoperatively, *p* < 0.001. The median of all airway measures improved however with very wide range. Subjects with the smallest amounts of constriction relief and/or gain in airway patency presented with least improvement in OSA-18. New airway measures show strong correlation with changes in OSA-18 (*ρ* = 0.44 to 0.71) whereas conventional measures showed very weak correlation (*ρ* = −0.04 to 0.37).

**Conclusions:**

Using point-based analyses, new airway measures better explained changes in clinical symptoms compared to conventional measures. Airway patency gained by at least 150% and constriction relief by at least 15% showed marked improvement in OSA-18 by 40–55%, after surgery in the tested cohort.

**Electronic supplementary material:**

The online version of this article (doi:10.1186/s40463-017-0204-4) contains supplementary material, which is available to authorized users.

## Background

The role of anatomical obstruction (adenoid, tonsillar, and nasal turbinates hypertrophy, deviated septum, and tongue position) on orthodontic changes and abnormal craniofacial growth are of continuous interest in the otolaryngology and orthodontic literature [1–3]. Controversies exist regarding the aetiology of paediatric sleep disordered breathing (SDB), but most accept anatomical obstruction of the upper airway as the most common cause. Adenotonsillar hypertrophy is considered the most important anatomic cause of such constriction thus prompting the American Academy of Paediatrics' recommendation of adenotonsillectomy (AT) as first line of treatment [4]. However, AT is not as effective as previously thought with failure rates as high as 54% in high-risk groups or comorbidities [5–7]. Information on underlying pathophysiologic mechanisms leading to residual SDB are limited. Only one study quantified volumetric changes using MRI in the paediatric upper airway with OSA after AT in which an association between residual adenoid tissue and low success rate of AT by means of polysomnography was found [7].

With its low radiation dose relative to multi-detector CT, cone beam CT (CBCT) provides insights to the anatomical anomalies found along the upper airway and craniofacial disproportions and has been used to measure anatomic airway changes with surgical and dental appliance treatment for adult SDB/OSA [8]. However, significant drawbacks were related to the questionable accuracy of the reconstructed upper airway 3D models, lack of clinical correlation with CBCT measurements, and the use of global non-specific airway measure such as volume, linear, and cross-sectional area measurements [8, 9]. Unlike global measures, point-based analysis with color mapping better explained differences in 3D upper airway models generated from CBCT [10]. Prospective studies analyzing the upper airway, by means of CBCT, and correlating anatomical airway changes with surgical outcomes in the SDB pediatric population are lacking [8, 11]. The aim of this clinical pilot is twofold: to prospectively evaluate anatomical changes that occur in the upper airways before and after AT using 3D airway models from CBCT; and to evaluate whether changes in anatomical airway measures are reflected in the patient’s quality of life in a cohort of children/adolescents presenting with jaw disproportions and SDB symptoms.

## Methods

### Subjects

This study was approved by the Health Research Ethics Board at the University of Alberta. Thirteen consecutive non-syndromic children-adolescents with SDB symptoms were recruited from the Interdisciplinary Airway Clinic (IARC), Department of Dentistry, University of Alberta. This tertiary center receives referrals from dentists or orthodontists in which the main concern of the subjects is maxillary-mandibular jaw disproportions with potential secondary SDB symptoms. As such, not many subjects undergo surgery. Based on the interdisciplinary evaluation of orthodontist, paediatric respirologist/sleep medicine specialist, and otolaryngology surgeon, the subjects underwent nasal or pharyngeal surgery to remove anatomical obstructions. A validated Pediatric Sleep Questionnaire (PSQ-22) and overnight pulse oximetry can be used as screening tools to identify SDB when PSG is not feasible [12–14]. The diagnosis of SDB is based on the history of nocturnal symptoms for at least 12 months, physical examination, overnight pulse oximetry, and Pediatric Sleep Questionnaire (PSQ-22) [15]. All subjects completed PSQ-22 and OSA-18 quality of life questionnaires and underwent CBCT imaging, over-night pulse oximetry, awake nasoendoscopy & laryngoscopy and sleep endoscopy simultaneous with surgery. OSA-18 questionnaire and CBCT imaging were also completed after surgery. The aim was to recall subjects by 6 months post-operatively to allow sufficient time for tissues to stabilize.

### CBCT imaging

The scans were obtained using Next generation iCAT® (Imaging Sciences International, Hatfield, PA) with 0.3 mm voxel, 4 s of exposure, 120 kVp, and 5 mA. The field of view extended from the Nasion superiorly to the chin inferiorly, the tip of the nose anteriorly and the bodies of cervical vertebrae posteriorly. Acquisition of CBCT scans was based on orthodontic reasons where conventional radiography failed to provide adequate information (e.g. maxillary constriction, anteroposterior or vertical discrepancies in the maxilla or mandible, or asymmetry). These disproportions are believed to be contributing factors to the SDB symptoms in this cohort with the prospects of maxillary expansion or orthognathic surgeries in their longer treatment plan justifying the use of CBCT. The authors do not support the use of CBCT for the sole purpose of airway analysis.

### Upper airway analysis

The upper airway region of interest (ROI) included the nasopharynx, oropharynx, and the nasal cavity (inferior and middle nasal meatus) and extends from the anterior nares to the level of anterior-inferior point of the body of the third cervical vertebra (C3), details in Additional file 1: Figure S1. The ROI was segmented and reconstructed into 3D model (ASCII STL format) using a semi-automatic program *Segura©,* developed and validated at the University of Alberta [10]. Using Mimics® [Mimics 15.0, Materialise NV, Leuven, Belgium], pre- surgical (T1) and post-surgical (T2) CBCT image sets were registered for each subject based on a previously validated method using six anatomical landmarks [16]. The 3D airway models were then imported and registered onto the “fused” CBCT image volumes using 10-point registration followed by global registration which fine-tunes the 10-point registration. The registered 3D models were exported to 3-matic® [3-matic 7.0, Materialise NV, Leuven, Belgium], smoothed by a factor of 0.7 and its surface wrapped. The upper airway was then divided into nasal cavity (NS), nasopharynx (NP), and oropharynx (OP) for further analysis using three planes, details are provided in Additional file 1: Figure S2. Airway measurements were carried out in 3-matic® and consisted of:

1. *Conventional* measures, at T1 and T2: **Volume** (cm^3^) and **surface area** (cm^2^) of NS, OP, and NP and **Minimum cross-sectional area** ***MinXarea*** (mm^2^) in OP or NP, at T1 and T2. MinXarea in OP was identified manually as the smallest medio-lateral dimension on the coronal view, and in the NP as the smallest anterior posterior dimension on the sagittal view, followed by confirmation on the 3D model of the airway.
2. *New* measures, at T1 and T2: **airway constriction and patency** of each segment at T1 and T2. These represent point-based analysis, referred to as “wall thickness analysis” in 3-matic®. The software measures the distance of each triangular node, forming the 3D mesh of the airway model, to the nearest surface based on the normal vector of the triangle, details in Additional file 1: Figure S3. The resultant analysis provides minimum, maximum, mean, median, standard deviation, and interquartile range of all the distances travelled by all the triangles from one surface to the opposing. From a given histogram, the percentage of triangles that traveled a distance within a certain threshold set by the operator can be chosen. In this pilot, distances <4 mm in the pharyngeal airway represent potential areas of **constriction**. Distances >10 mm in pharyngeal airway were considered areas of **patency**. These cut-off numbers were estimations by expert radiologist based on the CBCT radiographic appearance of the pharyngeal airway
3. Part Comparison analysis of each segment, T2-T1: This tool was previously described [10, 16] and represents point-based analysis to assess the changes in 3D airway models between T1 and T2 to produce a color map. A threshold was set between 4 and 10 mm such that areas marked in green represent tissue changes <4 mm, orange-yellow represent changes between 4 and 10 mm, and areas marked in red represent changes over 10 mm, from T1 to T2.

Conventional and new airway variables are measures before and after surgery, i.e. at T1 and T2, whereas part comparison analysis provided the color map of 3D airway model at T2 subtracted from T1.

### Statistical analysis

Statistical analysis was performed using IBM SPSS® [IBM SPSS Statistics 22.0, Armonk, NY]. Means (±standard deviations) are reported for normally distributed variables. For non-parametric variables, median and quartile range marking 25% deviation on each side of the median were reported as: median (lower *Q1*- upper *Q3* quartiles). For paired comparisons between T1 and T2 evaluations, Wilcoxon signed-rank test was used. To assess correlation between new and conventional airway measures with changes in OSA-18, Spearman Rho (ρ) correlation coefficients were completed. *P* value < 0.05 was considered significant.

## Results

Thirteen subjects were initially included in this pilot however one was excluded due to significant error in neck flexion and tongue positioning thus impacting the pharyngeal dimensions, where the subject received palatine tonsillectomy. The data of the remaining 12 was not normally distributed with few outliers, except for NS dimensions (normally distributed). Median (quartile range Q1-Q3) and other non-parametric tests were therefore used in this study, unless specified otherwise.

### Demographic/Clinical information

The mean age of the 12 subjects, 8 female and 4 male, was 8.8 ± 2 years. The mean recall period was 7 ± 1.5 months (range 4–9 months). Demographic, clinical, sleep naso-endoscopy, and type of surgery are summarized in Table 1. Of the 12, the mean BMI was 18.7 ± 3 (4 overweight or obese) and two had allergy and asthma. All subjects presented with short anterior cranial base, Sella-Nasion (SN) distance = 60.8 ± 3.1 mm and ranged from 55.5 to 64.9 mm. Seven (58.3%) presented with “long face syndrome”, three (25%) with narrow maxilla-high arched palate, and two (16.6%) with skeletal class III, i.e. prognathic mandible. At baseline, all subjects had sleep oximetry McGill score of 1 (i.e. normal or inconclusive of OSA) and mean PSQ-22 score of 0.50 ± 0.17. Nine (75%) had monopolar suction diathermy adenoidectomy with/without inferior turbinoplasty (microdebrider technique) and three (25%) had microdebrider assisted tonsillectomy (2 lingual and 1 palatine) with supraglottoplasty (cold steel technique), completed by the same ENT surgeon.

**Table 1:** Descriptive demographic, clinical, sleep endoscopy data per subject

	Age-Gender	BMI	PSQ proportion	Skeletal relationship	Allergy or Asthma	Sleep Naso-endoscopy Findings					Type of surgery	Change in OSA-18
						Chronic Rhinitis	Adenoids	Tonsils	Pharyngeal collapse	Additional notes		
1	9-F	16.8	50.0	Long face^a^ syndrome	No	moderate	<25%	<50% Palatine	None	-	Adenoidectomy	47%
2	10-F	16.5	55.0	Narrow maxilla & deep palate	No	moderate	>75%	<50% Palatine	AP collapse	-	Adenoidectomy	41%
3	12-F	19.9	27.2	Long face syndrome	No	mild	>75%	<50% Palatine	None	-	Adenoidectomy	45%
4	8-F	18.3	61.9	Long face syndrome	No	severe	>50–75%	<50% Palatine, Lingual TH^b^	None	-	Adenoidectomy	51%
5	7-F	14.7	54.5	Long face syndrome	No	mild	<25%	>50% Palatine	AP^c^ collapse	Laryngomalacia	Tonsillectomy & supraglottoplasty	67%
6	9-F	17.9	46.2	Long face syndrome	Yes	mild	<25%	<50% Lingual TH	AP collapse	Previous TNA^d^	Lingual tonsillectomy & supraglottoplasty	34%
7	6-M	18.3	33.3	Long face syndrome	Yes	mild	<25%	<50% Palatine	AP collapse	-	Adenoidectomy & turbinoplasty	22%
8	8-F	22.2	82.3	Prognathic mandible	No	severe	>50–75%	<50% Palatine	AP collapse	-	Adenoidectomy	52%
9	7-F	17.2	62.5	Narrow maxilla & deep palate	No	moderate	<25%	<50% Lingual TH	None	Previous TNA	Lingual tonsillectomy & supraglottoplasty	−13%
10	12-M	26	33.3	Narrow maxilla & deep palate	No	mild	>50–75%	<50% Palatine	AP collapse	-	Adenoidectomy & turbinoplasty	41%
11	7-M	14.7	40.0	Prognathic mandible	No	none	>50–75%	<50% Palatine	None	-	adenoidectomy	55.5%
12	11-F	17.2	50.0	Long face syndrome	No	mild	>50–75%	<50% Palatine	None	-	adenoidectomy	33%

^a^ Long face syndrome: narrow maxilla, high arched palate, retrognathic mandible with clockwise rotation. ^b^ *Lingual TH* lingual tonsil hypertrophy. ^c^ *AP collapse* antero-posterior collapse. ^d^ *TNA* tonsillo-adenoidectomy

### Quality of life

**Fig. 1:**
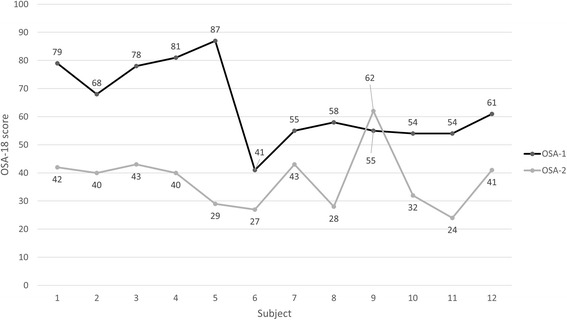
Scatter plot of OSA-18 scores before and after surgery per subject. Subjects 6 and 7 show smallest improvement whereas subject 9 presents with worsening symptoms

The median and quartile range for OSA-18 scores at baseline *T1* was 64.2 (54.7–79.5) and postoperatively *T2* was 37.6 (28.7–43). The total OSA-18 and sub-domain scores at T1 and T2 are summarized in Table 2. Subject 9 revealed worsening OSA-18 scores and subjects 6 and 7 presented with the smallest improvements in OSA-18 scores, Fig. 1.

**Table 2:** Average scores, median (Q1–Q3), for per- and post-operative OSA-18 questionnaires

	T1	T2	Score difference T1–T2		*P*-value^a^
			N	%	
Sleep disturbance	17 (12–22)	8 (7.5–10.5)	7.5 (4–10.7)	43.4 (33.3–61)	0.005
Physical suffering	16 (9.5–17.2)	10 (7–11.2)	5 (2.3–6.5)	34.8 (28.1–48)	0.05
Emotional Distress	11.5 (8.8–14.3)	7.5 (4–9.5)	3.5 (0.8–6.5)	36.6 (6.2–53.1)	0.03
Daytime problems	9 (6.8–16.5)	5.5 (4–9.7)	2.5 (0.8–7.7)	26.8 (15–47.8)	0.05
Caregiver Concern	14 (8.8–19)	5.5 (4–8.5)	7 (1–11.7)	43.9 (20–71.4)	0.005
Total score	64.2 (54.7–79.5)	37.6 (28.7–43)	25 (14.5–36.5)	40.9 (30.3–49.6)	<0.001

^a^ Wilcoxon signed Rank test

### Airway measurements

None of the subjects presented with significant nasal septum deviations. Generally, changes in NS dimensions from T1 to T2 were not statistically significant using paired *t*-test, mean volume of the NS was 11.2 ± 3 cm^3^ at T1 and 12.1 ± 3.1 cm^3^ at T2 (*p*-value = 0.07).

**Fig. 2:**
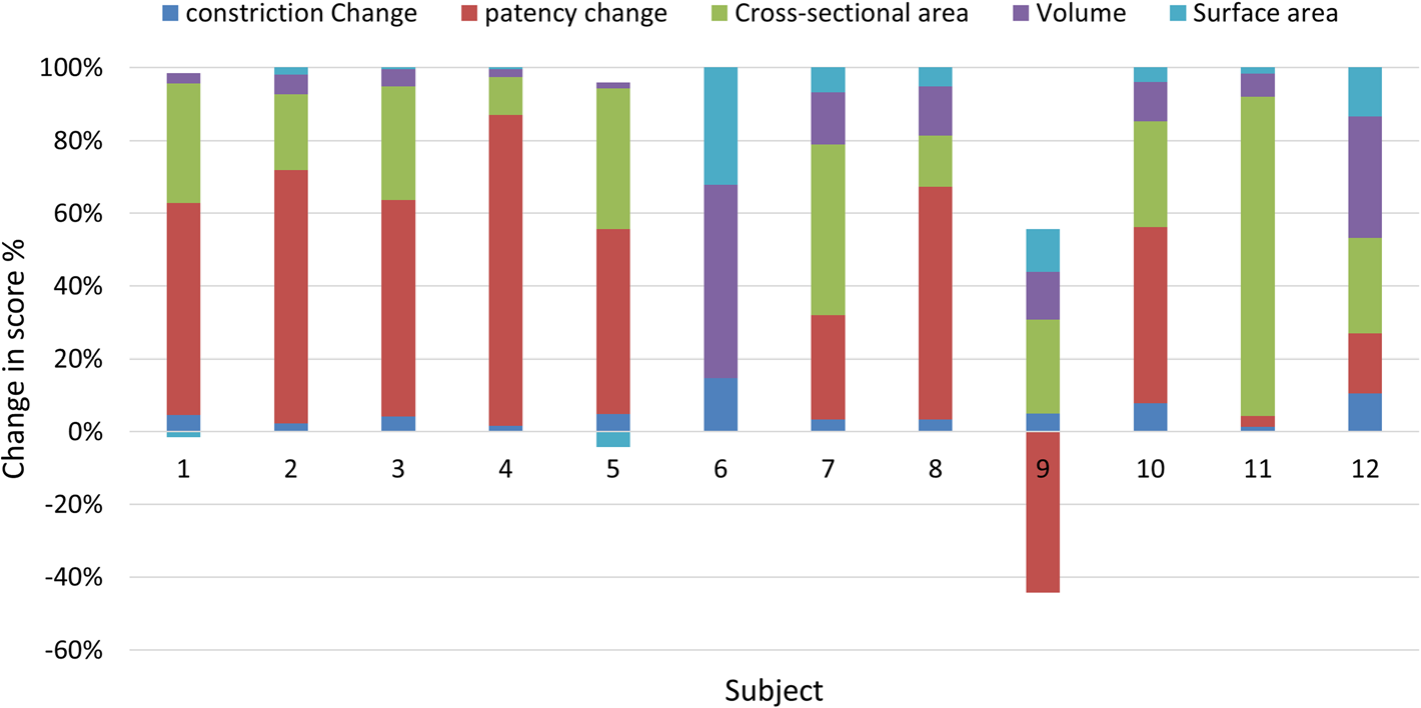
Stacked Bar Histogram of conventional airway measures specific to surgical area per subject. Conventional airway measures show considerable variability

Median changes in conventional and new airway measures specific to the area of surgery, i.e. NP for adenoidectomy and OP for tonsillectomy are presented in Table 3. Overall, the median of all airway measures in the surgical area showed improvement after surgery however with very wide range. Changes in conventional and new airway variables per subject are presented in Fig. 2. Using new airway measures, subjects #6, 7, and 9 showed small amounts of constriction relief and gain in patency. Subject #9 had lost airway patency after lingual tonsillectomy by 75% and subject #6 had no change in airway patency.

**Table 3:** Average airway measurements specific to the surgical area

Airway measure	T1 Mean (minimum-maximum)	T2 Mean (minimum-maximum)	% Score difference T2-T1			*P*-value^b^
			Median (Q1-Q3)	Minimum	Maximum	
Volume (cm^3^)^c^	4.9 (1.8–10.4)	8.4 (2.2–18)	42.8 (24.8–141.4)	4.6	203.3	0.002
Surface area (cm^2^) ^c^	18.8 (1.9–29.3)	22.6 (2.7–38)	24 (5.2–41.9)	−12.3	81.8	0.02
MinX area (cm^2^) ^c^	1.1 (0.1–3)	2.7 (0.4–3.9)	164.6 (109.7–246.2)	0	2114.2	0.005
Airway constriction <4 mm (%)	49 (26–92)	32.3 (22–68)	29 (13.1–46.1)^a^	7.7	64.1	0.002
Airway patency >10 mm (%)	3.1 (0–8)	14 (1–31)	216.6 (67–774.5)	−75	1450	0.006

^a^T1–T2

^b^Wilcoxon signed rank

^c^ These measures are specific to the area of surgery; NP for adenoidectomy and OP for tonsillectomy

**Fig. 3:**
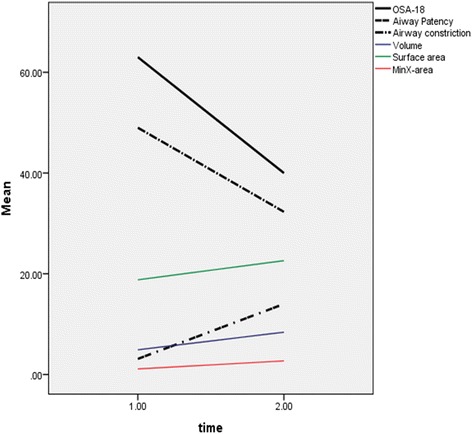
Line chart of median airway measures and OSA-18 scores at T and T2. The degree of change from T1 to T2 in the median airway constriction and patency is very similar to that of OSA-18

Conventional airway measures show low correlation with quality of life outcome (OSA-18): Spearman Rho (ρ) = 0.37 for MinXarea, 0.16 for volume, and − 0.13 for surface area. Whereas new airway measures show moderate to strong correlation: Spearman Rho = 0.44 for constriction and 0.71 for patency. Correlation between OSA-18 and all airway measures is presented in Fig. 3. New airway measures show very strong (ρ = 0.69–0.86) and significant correlations with conventional measures such as volume and MinXarea, details in Additional file 1: Table S1.

**Fig. 4:**
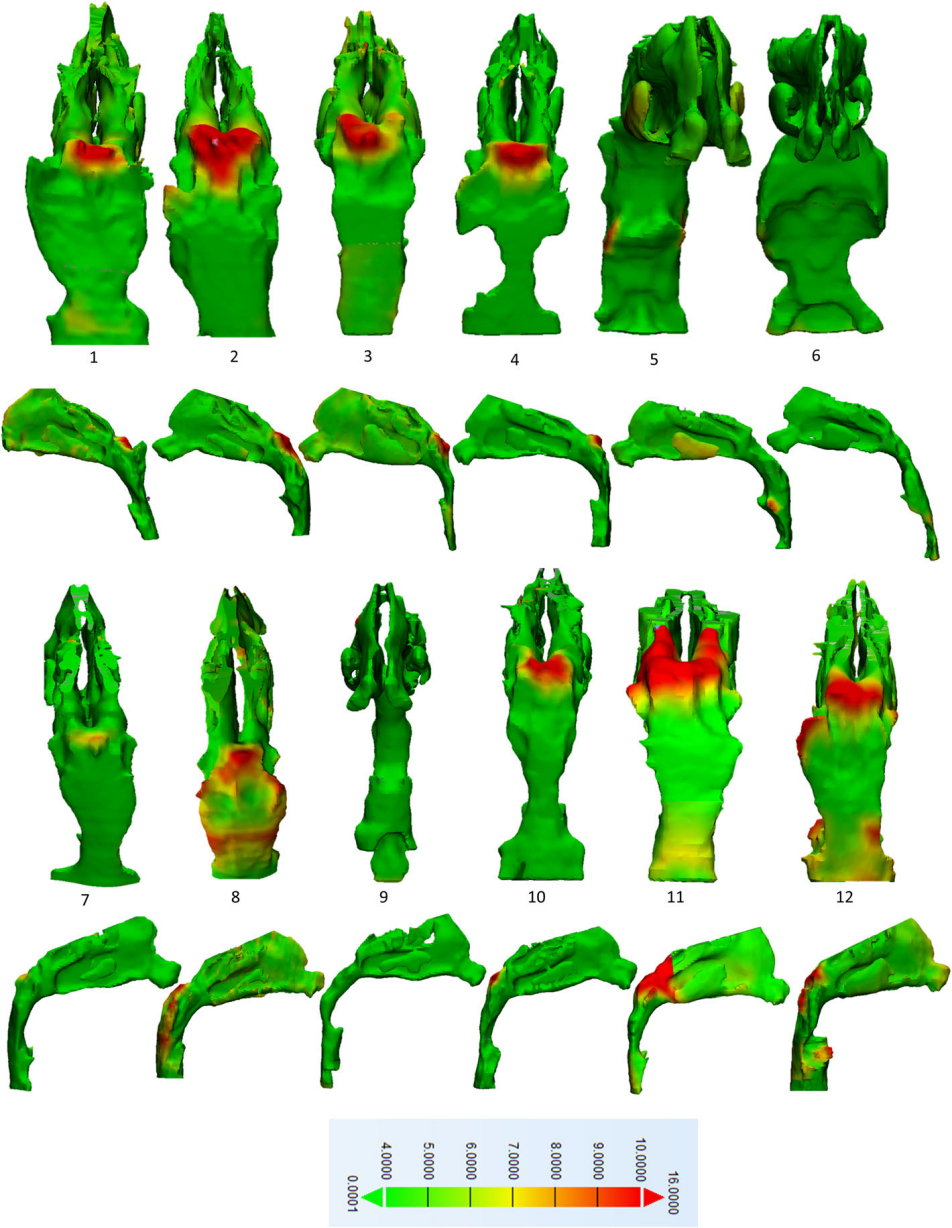
Part analysis T2-T1 of subjects 1 though12. Tissue changes after surgery <4 mm are marked by *green* and changes >10 mm are marked in *red*. Subjects 5, 6, and 9 received tonsillectomies whereas the remainder received adenoidectomy

Part comparison analysis (T2–T1) depicts changes in airway models specific to surgical areas, Fig. 4.

## Discussion

In this clinical pilot, we present the use of 3D models of the upper airways reconstructed from CBCT to assess surgical outcomes in 12 children-adolescents presenting with SDB symptoms and jaw disproportions in the interdisciplinary airway clinic.

All 12 subjects presented with short anterior cranial base, SN = 60.8 ± 3.1 mm, similar to a recent study [17] of OSA children (mean age 9) where mean SN was 61.5 ± 3.4 mm. Compared to normative data published for 10 year olds mean SN = 63.9 ± 2.6 mm [18] and 70.8 ± 2.9 mm [19], children with OSA had shorter anterior cranial base lengths, highlighting the interplay between craniofacial form and airway space.

Ideally, full polysomnography (PSG) would be used to diagnose SDB and assess surgical outcome however, it is expensive, time consuming, labor intensive, and very few Canadian institutions can routinely use full PSG. Although sleep pulse oximetry did not rule out SDB (McGill score = 1) at baseline, the PSQ-22 scores were over the published cut off (≥0.33) [12] for 10 subjects out of 12 indicating high risk of pediatric SDB.

Similar to other studies, the surgical outcomes in this study were assessed using the OSA-18 quality of life questionnaire [13, 20, 21]. Overall, the impact of SDB-symptoms on patients’ quality of life reduced after surgery in total OSA-18 score and its subdomains, Table 2. Median total OSA-18 score changed from moderate impact to low. This is based on using the cut off of 60 where total score <60 is low, 60–80 is moderate, and >80 is severe impact on quality of life [22]. Several studies reported changes in OSA-18 in children post AT or tonsillectomy, the mean baseline OSA-18 ranged from 61.1 to 77.6 and the range of mean OSA-18 postoperatively was 32.5 to 41 [23–26]. Our cohort presents with similar values at T1 and T2. Subjects 6 and 7 showed the least amount of improvement whereas subject 9 reports worsening of symptoms marked by higher OSA-18 scores in T2 (OSA-18 score was 55 and increased to 62 post-surgically), Fig. 1. After surgery, the parent of subject 9 reports development of swallowing difficulties, aggressive behavior, and difficulties in waking up in the morning. This is a subtle sign of the possible neuro-behavioral and reduced neuromuscular tone contributing factors in the realm of pediatric SDB. Of note, subjects 6 and 9 had lingual tonsillectomies and subjects 6 and 7 were siblings with asthma and allergy.

This pilot is the first to utilize 3D models generated from CBCT to analyze the upper airways pre- and post AT in pediatric SDB. None of the 12 subjects presented with significant nasal septum deviation or nasal constriction. Nose (NS) volume did not change from T1 to T2 even in the two subjects that underwent turbinoplasty (#6 and 10) possibly due to mucosal thickening at T2.

Overall, there was significant improvement in airway dimensions after surgery in all 12 subjects however with wide range, Table 3. Subjects 1 and 5 showed the smallest changes in volume and surface area, Fig. 2. Similar to our results, Nandalike et al. [7] reported volume increases in NP (from 2.9 ± 1.3 to 4.4 ± 0.9 cm^3^) and in OP (from 3.2 ± 1.2 to 4.3 ± 2.0 cm^3^). In their study, 27 obese children with OSA underwent PSG and MRI and the volumes of the NP, OP, adenoids, tonsils, and tongue were measured pre and post AT. MinXarea increased for all subjects except for #6; remained unchanged after surgery. This highlights the deficiency in using MinXarea which focuses on one slice (through the adenoidectomy site) and neglects the entire airway.

Volume and surface area are nonspecific measures because local changes or differences are overlooked. CBCT point-based analysis was previously used to assess postsurgical changes in the craniofacial area [27, 28] and specifically in the upper airway [10, 16]. Using new airway measures, the mean relief of constriction was 29% and mean gain of patency by 216.6%, Table 3. In other words, tissues showing airway lumen narrowing <4 mm, marking potential sites of collapse, have reduced and areas with over 10 mm lumen patency have increased post-surgically. This however was not the case in subject 9 showing loss of previous patency by 75% and unchanged airway patency in subject 6, Fig. 2. Subjects 6, 7, and 9 show the least amounts of changes in airway constriction as well as OSA-18, Fig. 2. While airway constriction of subject #5 modestly improved by 14.3%, there was a large gain airway patency by 150% after palatine tonsillectomy and presented with the greatest improvement in OSA-18 score by 67%. Subjects 6 and 9 underwent lingual tonsillectomy with history of failed AT and thus already present with complexity in which lingual (T) was the last surgical resort. Hypertrophy of the lingual tonsils occurred in one third of children with persistent OSA and along with allergy and asthma present risk factors to residual SDB [4, 29, 30]. Subject 7 underwent (A) and presented with allergy, asthma, and family history of SDB (i.e. subject 6/sibling) all of which are risk factors to residual SDB [29, 30].

It appears that gaining airway patency beyond 150% and relieving constriction beyond 15% after surgery did not drastically change scores in the OSA-18; all subjects other than 6, 7, and 9 tend to “plateau” at 40–55% improvement in OSA-18 post-surgically, Figs. 1 and 2. This is suggestive of a possible threshold of surgical tissue changes beyond which it has low impact on changes in quality of life. This evidently needs to be verified with a larger sample size.

Only new airway parameters, constriction and patency, showed strong correlation with changes in OSA-18 There was moderate correlation between changes in OSA-18 and MinXarea and no-weak correlation with volume and surface area. This suggests that changes in new airway variables better represent the degree of changes in OSA-18 compared to conventional measures, Fig. 3.

When correlating conventional and new airway measures, there was strong correlation confirming that point-based analysis is supplemental to global/conventional measures yet is more explanatory as it takes into account the level (s) of narrowing throughout the entire 3D object i.e. the airway.

The amount and localization of tissues removed and airway space gained is illustrated in Fig. 4. Changes were more noticeable in adenoidectomy cases and least in subjects 6, 7, and 9. Overall, 16.5% of the tissues gained space over 4 mm in the 12 subjects, and 1.5% of tissues gained space beyond 10 mm.

Limitations to this pilot are related to the small sample size, heterogeneity of the surgeries included, and existing outliers severely limit options to statistical tests and hinder the *P*-values reported. However, the intention of this level-I diagnostic study was to test feasibility of using new airway measures to assess surgical outcomes.

Future studies with controls and larger sample size will allow rigorous statistical analyses such as regression and discriminant analyses. Ultimately multiple clinical and imaging variables can be tested to provide a prediction model.

## Conclusions

This pilot is the first to prospectively evaluate anatomical changes in the upper airways after adenoidectomy or tonsillectomy using accurate 3D airway models from CBCT with meaningful tools of analysis. In this cohort, it was evident that:

- New airway measures, *airway patency and constriction*, strongly correlated with quality of life measure (OSA-18) and better explained low scores after surgery.
- Airway patency and constriction also strongly correlated with conventional measures and proved more explanatory.
- Airway patency gained by at least 150% and constriction relief by at least 15% showed marked improvement in OSA-18 by 40–55%, after surgery.

## Additional file

Additional file 1: Supplemental Material, **Table S1.** Correlation between new and traditional airway measures. **Figure S1.** Sections of the upper airway. **Figure S2.** Three planes used to section the upper airway models. **Figure S3.** New airway measures (airway constriction and patency). (DOCX 4915 kb)

## Abbreviations

A or T: Adenoidectomy or tonsillectomy
BMI: Body mass index
CBCT: Cone beam computed tomography
MinXarea: Minimum cross-sectional area
NP: Nasopharynx
NS: Nasal cavity
OP: Oropharynx
OSA: Obstructive sleep apnea
PSG: Polysomnography
PSQ: Pediatric sleep questionnaire
ROI: Region of interest
SDB: Sleep disordered breathing
SN: Sella-Nasion
STL: STereoLithography
