# Supplementary material for: Using cone beam CT to assess the upper airway after surgery in children with sleep disordered breathing symptoms and maxillary-mandibular disproportions: a clinical pilot
Source: J Otolaryngol Head Neck Surg. 2017 Apr 11;46:31. doi: 10.1186/s40463-017-0204-4 (PMC5387252; doi:10.1186/s40463-017-0204-4)
Supplement: Additional file 1: — Supplemental Material, Table S1. Correlation between new and traditional airway measures. Figure S1. Sections of the upper airway. Figure S2. Three planes used to section the upper airway models. Figure S3. New airway measures (airway constriction and patency). (DOCX 4915 kb) [file 40463_2017_204_MOESM1_ESM.docx]

**Additional Material**

1 table and 3 figures

| **Additional Table 1: Correlation between new and traditional airway measures** | | | |
| --- | --- | --- | --- |
| **New Measures** | **Traditional Measures** | | |
|  | **Change in MinX-sectional area %** | **Change in Volume %** | **Change in surface area %** |
| **Relief in Airway constriction %** | 0.86* | 0.77** | 0.69* |
| **Gain in Airway Patency %** | 0.88* | 0.55 | 0.24 |
| **Correlation is significant at the 0.01 level (2-tailed).  *Correlation is significant at the 0.05 level (2-tailed). | | | |


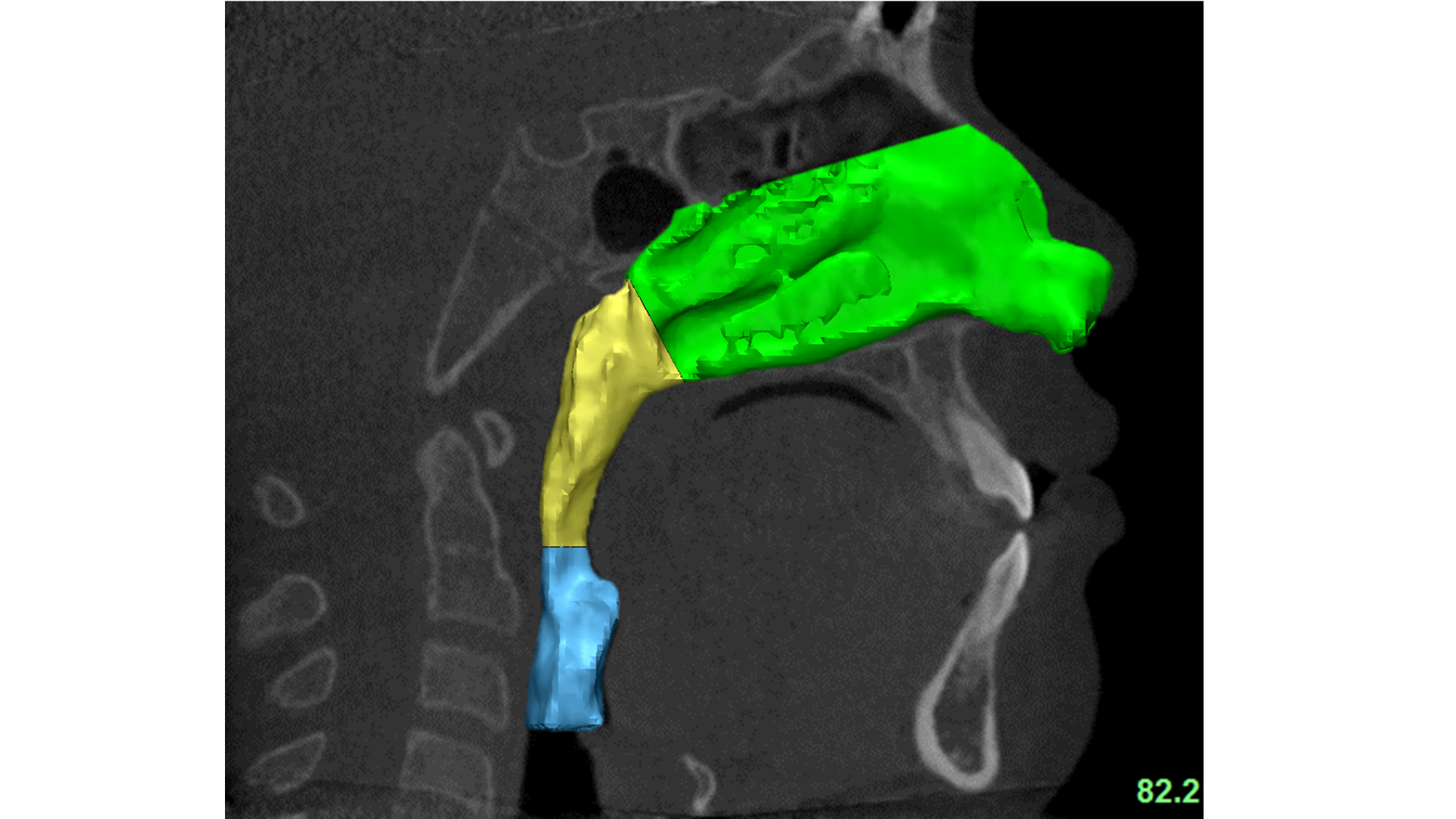


**Additional Figure 1:** Sections of the upper airway. Sagittal CBCT image showing Nose (NS) in green, Nasopharynx (NP) in yellow, and Oropharynx (OP) in blue.


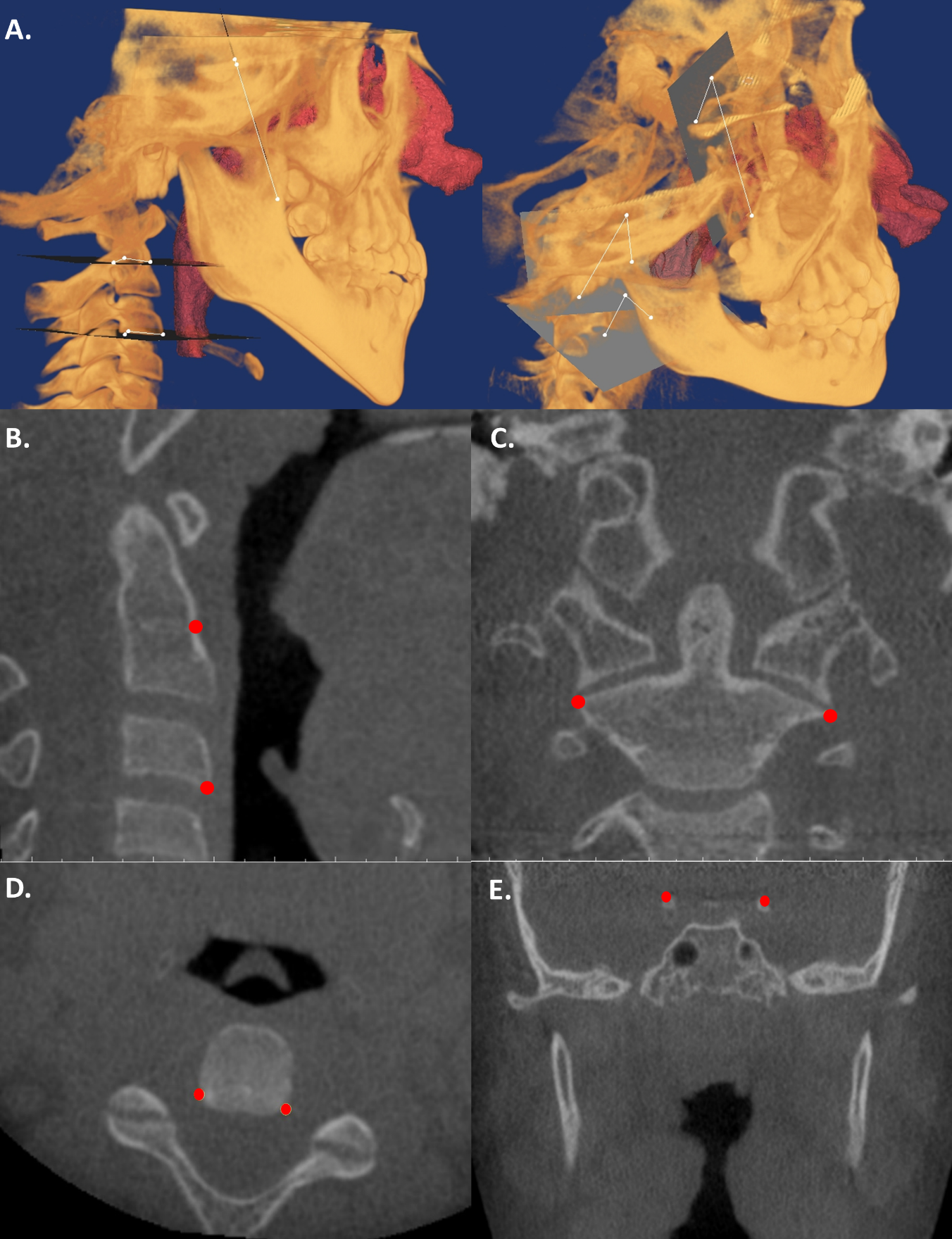
**Additional Figure 2:** Three planes used to section the upper airway models. (A) 3D rendering of skull and airway showing the 3 planes. (B) Sagittal CBCT with anterior points of 2^nd^ and 3^rd^ planes: anterior-inferior base of odontoid and anterior-inferior body of C3. (C) Coronal CBCT with posterior points of 2^nd^ plane: anterior-lateral points of C2 pedicles. (D) Axial CBCT with posterior points of 3^rd^ plane: posterior-inferior-lateral points of body C3. (E) Coronal CBCT with posterior points of 1^st^ plane: anterior clinoid processes.


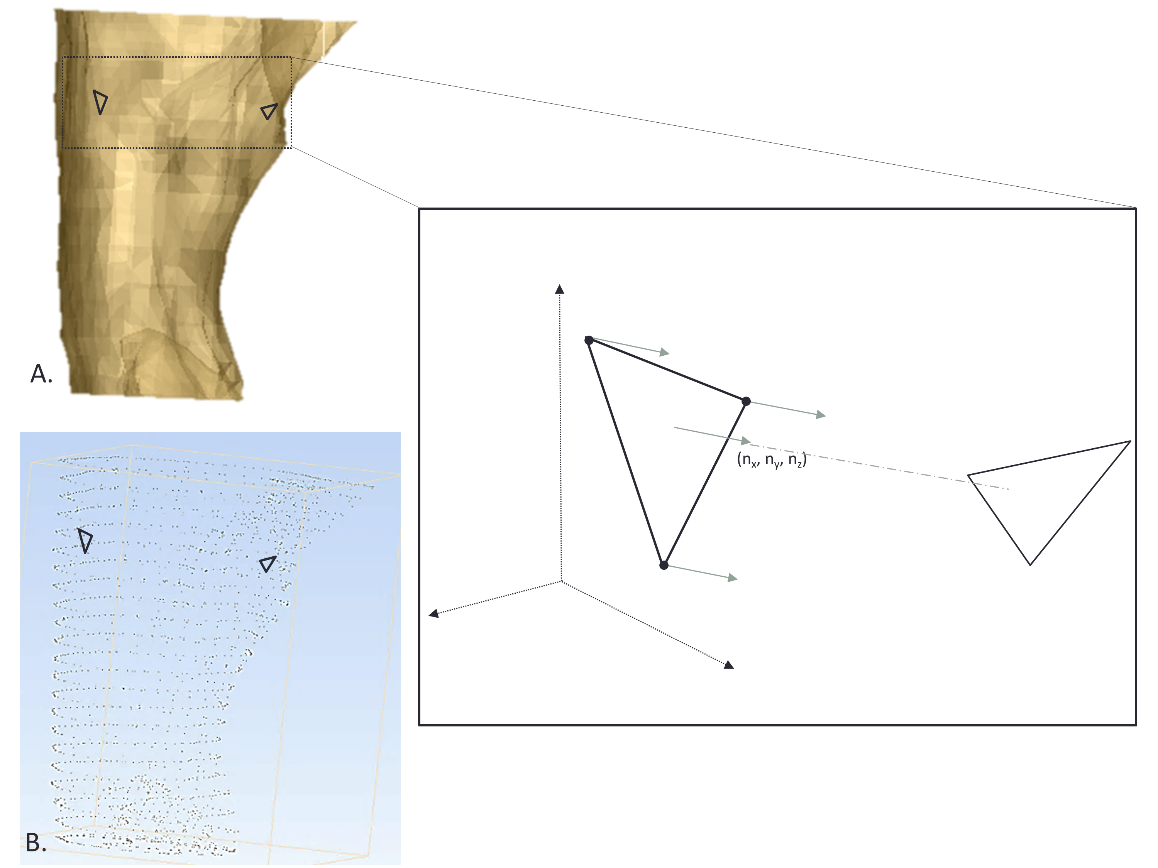


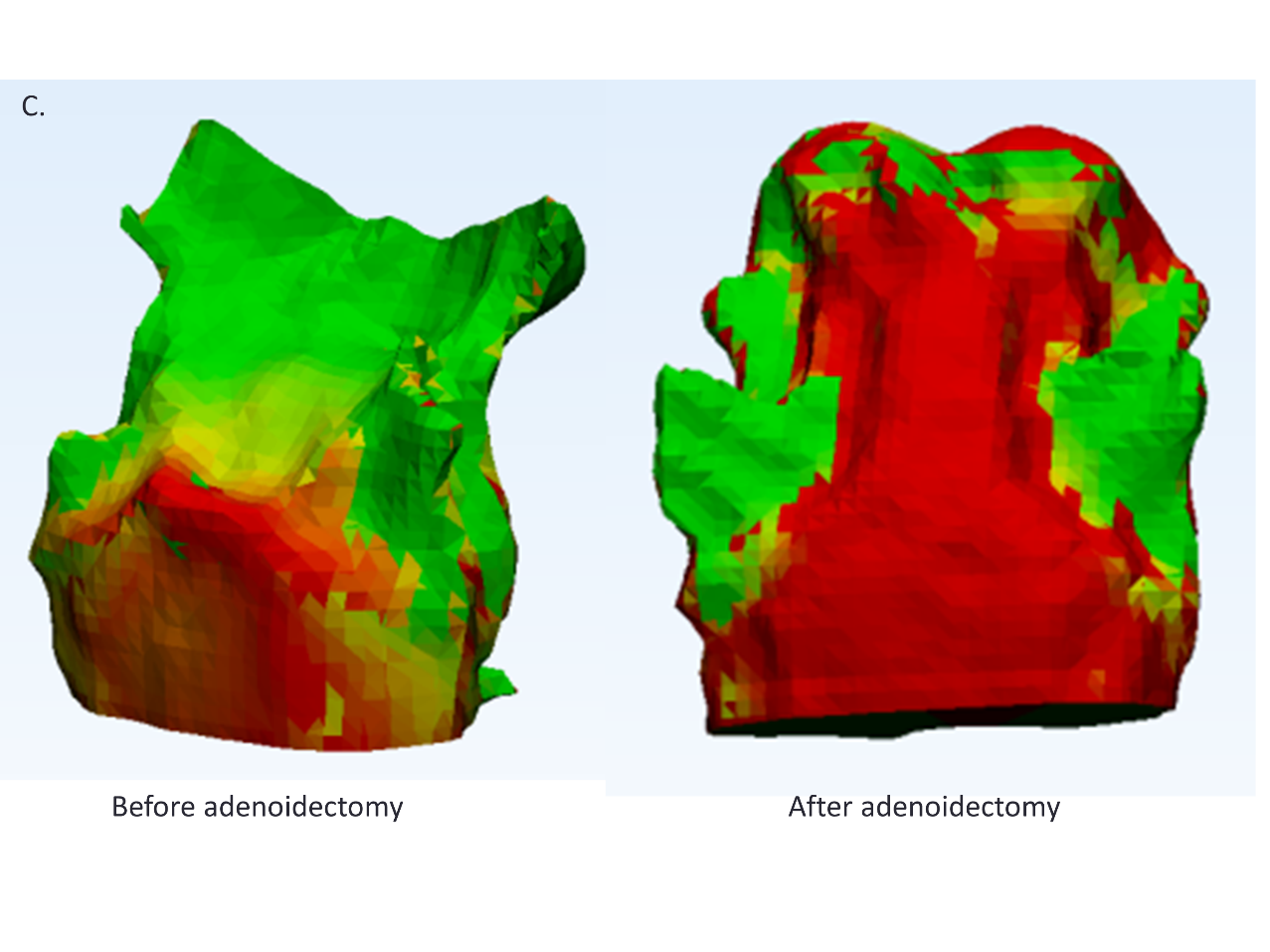


**Additional Figure 3:** New airway measures (airway constriction and patency). (A) 3D model of the oropharynx and (B) triangle nodes forming the model with two sampled triangles: inset shows vector of one triangle to the nearest triangle on opposing surface. (C) Example of color map where triangles in green represent distances <4mm (i.e. area of constriction at adenoids) and in red > 10 mm (i.e. area of patency increased after adenoidectomy)
